# Clinical Isolates of *Acinetobacter* spp. Are Highly Serum Resistant Despite Efficient Recognition by the Complement System

**DOI:** 10.3389/fimmu.2022.814193

**Published:** 2022-01-31

**Authors:** Michal Magda, Serena Bettoni, Maisem Laabei, Derek Fairley, Thomas A. Russo, Kristian Riesbeck, Anna M. Blom

**Affiliations:** ^1^ Protein Chemistry, Department of Translational Medicine, Lund University, Malmö, Sweden; ^2^ Department of Biology and Biochemistry, University of Bath, Bath, United Kingdom; ^3^ Department of Microbiology, Belfast Health and Social Care Trust, Belfast, United Kingdom; ^4^ Veterans Administration Western New York Healthcare System, Department of Medicine, Jacobs School of Medicine and Biomedical Sciences, University Buffalo, Buffalo, NY, United States; ^5^ Clinical Microbiology, Department of Translational Medicine, Lund University, Malmö, Sweden

**Keywords:** *Acinetobacter baumannii*, capsule, complement, membrane attack complex, phagocytosis

## Abstract

Gram-negative bacteria from the genus *Acinetobacter* are responsible for life-threating hospital-related infections such as pneumonia, septicemia, and meningitis, especially in immunocompromised patients. Worryingly, *Acinetobacter* have become multi- and extensively drug resistant (MDR/XDR) over the last few decades. The complement system is the first line of defense against microbes, thus it is highly important to increase our understanding of evasion mechanisms used by *Acinetobacter* spp. Here, we studied clinical isolates of *Acinetobacter* spp. (n=50), aiming to characterize their recognition by the complement system. Most isolates tested survived 1 h incubation in 30% serum, and only 8 isolates had a lower survival rate, yet none of those isolates were fully killed. Intriguingly, four isolates survived in human whole blood containing all cell component. Their survival was, however, significantly reduced. Flow cytometry analyses revealed that most of the isolates were detected by human IgG and IgM. Interestingly, we could not detect any significant concentration of deposited C1q, despite observing C4b deposition that was abolished in C1q-deficient serum, indicating transient binding of C1q to bacteria. Moreover, several isolates were recognized by MBL, with C4b deposition abolished in MBL-deficient serum. C3b was deposited on most isolates, but this was not, however, seen with respect to C5b and formation of the membrane attack complex (MAC), indicating that many isolates could avoid complement-mediated lysis. India ink staining showed that isolates were capsulated, and capsule thickness varied significantly between isolates. Studies performed on a wild-type strain and capsule mutant strains, demonstrated that the production of a capsular polysaccharide is one mechanism that mediates resistance to complement-mediated bactericidal activity by preventing MAC deposition and lysis. Our data showed that most clinical *Acinetobacter* spp. isolates are highly serum resistant despite being efficiently recognized by the complement system.

## Introduction

Bacteria from the genus *Acinetobacter* are causing life-threating infections such as pneumonia, septicemia, and meningitis. Among *Acinetobacter* spp., *Acinetobacter baumannii* has emerged as the most clinically important subspecies. The other subspecies like *A. pittii, A. lwoffii* and *A. ursingii* are, however, also clinically relevant, and often isolated from hospitalized patients. Over the last few decades, *Acinetobacter* have become an increased threat to human health because of increasing multi- and extensive drug resistance (MDR/XDR) among this species (1–5). At present, antimicrobial resistance (AMR) is one of the top 10 threats to global health (6–9). Consequently, the World Health Organization (WHO) have identified ‘priority pathogens’ where *A. baumannii* ranks as Priority 1, for which the development of new antibiotics and treatment options are urgently required (10). Immunocompromised hosts and patients treated at intensive care unit (ICU) are particularly vulnerable to *A. baumannii*. During the current COVID-19 pandemic, an increased number of ICU patients suffering from SARS-CoV-2 and extensive use of antibiotics has resulted in a higher number of secondary infections caused by MDR *A. baumannii* (11). Co-infections with *A. baumannii* have significantly worsened the outcome for COVID-19 patients resulting in a higher mortality, compared to patients without bacterial co-infections (12–14). Besides MDR/XDR, *Acinetobacter* spp. are known to express several virulence factors that help them to survive in the environment, but most importantly to evade host immune system during infection (15). The polysaccharide capsule is one of the virulence factors that protects *Acinetobacter* spp. from unfavorable environmental conditions as well as from phagocytosis and direct lysis by complement or killing by antimicrobial compounds (15–18).

As a part of the innate immune system, the complement system plays a significant role in protection from bacterial invasion (19). Complement activation leads to the formation of membrane attack complex (MAC), which deposited into bacterial outer membrane causes direct lysis of the pathogen. Apart from the formation of MAC, deposition of C3b targets bacteria for efficient uptake by phagocytic cells, while release of anaphylatoxins C3a and C5a attracts cells to the infection site (20). Interestingly, *Acinetobacter* spp. can escape from complement and phagocytosis using CipA, an outer membrane protein, which degrades fibrinogen and opsonin C3b (21). Furthermore, bacteria can be coated with immunoglobulins and the Fc domain of bound immunoglobulins can be consequently recognized by Fc receptors on phagocytes (22, 23). Indeed, some previous studies showed that neutrophils and tissue macrophages, contribute to the killing of *Acinetobacter* cells by releasing cytokines, reactive oxygen species (ROS) and forming neutrophil extracellular traps (NETs) (24, 25). However, it was also showed that *Acinetobacter* spp. successfully escape from the uptake by neutrophils and from NETs (26, 27). Additionally, impairment of neutrophils function resulted in enhanced systemic infection (28). Previous studies indicated that macrophages are important during early stage of infection, where they are responsible for the active uptake of bacteria and release of proinflammatory cytokines (24).

Complement must be tightly regulated to prevent unwanted activation and lysis of host cells. The main soluble inhibitors are C4b-binding protein (C4BP), which regulates classical and lectin pathways, and Factor H (FH), which inhibits the alternative pathway. Many bacteria developed mechanisms to escape complement by hijacking complement inhibitors thus avoiding recognition and clearance by the immune system (29). Pathogens often bind FH (30, 31) or C4BP (32, 33) to stay protected from activation of complement and lysis by MAC. One report suggested that *A. baumannii*, using the outer membrane porin OmpA, binds FH, which contributes to its serum resistance (34). Interestingly, OmpA protein was also suggested as target for the antimicrobial peptide LL-37 (35). LL-37 binds *A. baumannii* in a dose-dependent manner, decreasing survival, motility, and adhesion of the pathogen.

*Acinetobacter* infections are becoming an important health issue worldwide and knowledge gap exists regarding activation of the complement system during *Acinetobacter*-dependent infection. Therefore, we screened the susceptibility to complement-mediated bactericidal activity by choosing a series of various clinical isolates. We found that most isolates were well recognized by complement, but despite this, bacteria were complement resistant and not lysed by MAC.

## Materials And Methods

### Bacterial Strains and Growth Conditions

To compare *Acinetobacter* spp. having various virulence properties, several different clinical isolates of *Acinetobacter* spp. were selected for the study. Since *A. baumanii* is the most important and virulent subspecies causing human disease, most strains chosen were *A. baumanii*. In addition, *A. pittii, A. nosocomialis* and *A. calcoaceticus* can also cause infections in human and are often isolated together with *A. baumannii* as the Abc complex. In contrast, *A. lwofffii* and *A. johnsonnii* are mainly found on the skin and in the environment but may also cause disease in immunocompromised hosts. Clinical isolates were obtained from Royal Victoria Hospital, Belfast Health & Social Care Trust, Belfast (Northern Ireland) and Clinical Microbiology, Laboratory Medicine Skåne, Lund (Sweden). Bacteria were isolated from hospitalized patients and their identity verified using matrix assisted laser desorption ionization-time of flight mass spectrometry (MALDI-TOF MS; Bruker). *Acinetobacter baumannii* ATCC 19606 strain was purchased from the Culture Collection University of Gothenburg (CCUG) (Sweden) (36–38) for a total of 50 *Acinetobacter* strains used for the study. Detailed information regarding each isolate including antibiotic resistance can be found in **Supplementary Table 1**. *Acinetobacter baumannii* 307-0924 wild-type strain and isogenic mutant derivatives (**Supplementary Table 2**) were described previously (16). For all experiments included in the manuscript bacteria were grown using the same procedure: bacteria were grown on blood agar plates overnight. Following overnight incubation, bacteria were subcultured onto fresh blood agar plates for an additional 5 h. All strains were cultured at 37°C, whereas *A. johnsonii* strains were cultured at room temperature. Kanamycin (50 µg/mL) (Duchefa Biochemie, K0126) and carbenicillin (200 µg/mL) (Sigma-Aldrich, C1389) were used for selection of mutant isolates. *Moraxella catarrhalis* RH4 and *Haemophilus influenzae* 3655 were grown overnight on chocolate agar plates at 37°C with 5% CO_2_. Following day, bacteria were subcultured onto fresh chocolate agar plates and incubated for an additional 5 h. All isolates were stored at -80°C in Brain Heart Infusion broth (Merck, 110493) containing 30% glycerol.

### Proteins and Antibodies

C1q protein was purified from human plasma as described (39). Recombinant *Ornithodoros moubata* complement inhibitor (OmCI) protein was expressed in *E. coli* as described (40). C1-inhibitor (C1-INH) protein (Berinert^®^) was purchased from CSL Behring. Compstatin was acquired by courtesy of John D. Lambris, University of Pennsylvania, PA (USA) (41). Cytochalasin D was purchased from Sigma-Aldrich (C8273).

For the flow cytometric analyses of the complement deposition, the following antibodies were used: pAb rabbit anti-human C1q (Dako, A0136), pAb rabbit anti-human C3d (Dako, A0063), pAb rabbit anti-human C4c (Dako, Q0369), mAb mouse anti-human iC3b (Quidel, A209), pAb rabbit anti-human C5b (42), mAb mouse anti-human C9 neoantigen (Hycult Biotech, HM2167). For the detection the following secondary antibodies were used: pAb donkey f(ab)_2_ anti-rabbit IgG conjugated to Alexa Fluor 647 (Jackson ImmunoResearch, 711-606-152) and pAb goat anti-mouse IgG conjugated to Alexa Fluor 488 (Invitrogen, A32723). Binding of human Igs was detected using pAb goat anti-human IgG conjugated to Alexa Fluor 488 (Invitrogen, A11013) and pAb goat anti-human IgM conjugated to Alexa Fluor 488 (Thermo Fisher, A-21215). Binding of human complement inhibitors was detected using biotinylated, mAb anti-Factor H [MRC OX24, (43)] and mAb anti-C4BP [MK104, (44)] antibodies. For the detection of biotinylated antibodies, streptavidin conjugated to Alexa Fluor 647 (Life Technologies, S32357) was used. For the detection of polysaccharide capsule, mAb mouse a-K1 serotype IgM [MAb 13D6, (16)] and mAb mouse control IgM (ImmunoTools, 21275051S), with secondary pAb goat anti-mouse IgM (µ-chain) conjugated to Alexa Fluor 647 (Invitrogen, A21238) antibodies were used. For the flow cytometry assays, bacteria were stained with CellTrace Violet (Thermo Fisher Scientific, C34557) and minimum 20 000 events were examined.

### Normal Human Serum and Depleted Sera

Normal human serum (NHS) was obtained from the blood from 12 healthy volunteers who provided written consent according to the recommendations of the local ethical committee in Lund (Sweden; 2019/14) and the Declaration of Helsinki (45). NHS was collected as described (46) and stored at -80°C. NHS with 20 mM EDTA was used as a negative control for the complement activation experiments. Heat-inactivated normal human serum was obtained after incubation of NHS at 56°C for 30 min.

Human C1q-depleted serum was purchased from Quidel (A509) or prepared in house during the purification of C1q protein. Human IgG/IgM-depleted serum was purchased from Pel-Freez Biologicals (34010). Human MBL deficient serum was purchased from BioPorto Diagnostics (SER 103).

### Complement Deposition Assays

For the activation of all complement pathways bacteria were harvested, washed in PBS, and diluted in GVB^++^ buffer (5 mM Veronal buffer [pH 7.3], 140 mM NaCl, 0.1% gelatin, 1 mM MgCl_2_ and 5 mM CaCl_2_) to OD_600_ = 1. To study C1q deposition bacteria were incubated for 15 min at 37°C with 2.5% NHS. C1q-depleted serum (Quidel) was used as a negative control. For the deposition of C3b, C4b and MAC bacteria were incubated with 10% NHS or 10% NHS with 20 mM EDTA for 1 h at 37°C. Deposition of C4b in depleted sera was done in a similar fashion using in-house human C1q-depleted serum or MBL-depleted serum (BioPorto). In supplementary experiments, increasing concentration of NHS (2.5%, 10% and 30%) were used to detect C1q and C4b deposition, including the two gram-negative strains *M. catarrhalis* RH4 and *H. influenzae* 3655 as controls. To block complement activation, 0.5 mg/mL of C1-INH was used. After incubation, bacteria were washed with PBS and stained with anti-C1q, anti-C3d, anti-C4c and anti-C9 neoantigen antibodies for 30 min at RT. After washing with PBS, primary antibodies were detected with secondary donkey f(ab)_2_ anti-rabbit IgG Alexa Fluor 647 or goat-anti mouse IgG Alexa Fluor 488.

For the activation of alternative pathway bacteria were diluted in Mg-EGTA buffer (2.5 mM Veronal buffer [pH 7.3], 70 mM NaCl, 140 mM glucose, 0.1% gelatin, 7 mM MgCl_2_, and 10 mM EGTA) to OD_600_ = 1. Similarly, bacteria were incubated with 10% NHS or 10% NHS with 20 mM EDTA for 30 min at 37°C. After incubation, bacteria were washed with PBS and stained with anti-iC3b, anti-C3d anti-C5b and anti-C9 neoantigen antibodies for 30 min at RT. Subsequently, bacteria were washed, and primary antibodies were detected with secondary donkey f(ab)_2_ anti-rabbit IgG Alexa Fluor 647 or goat-anti mouse IgG Alexa Fluor 488 antibodies. Bound antibodies were measured using CytoFLEX multicolor flow cytometer (Beckman Coulter).

### Binding of Immunoglobulins to Bacteria

Bacterial cells were harvested, washed in PBS, and diluted in GVB^++^ buffer to OD_600_ = 1. For the binding of immunoglobulins, bacteria were mixed with 2.5% NHS or 2.5% IgG/IgM-depleted serum. After 15 min incubation at 37°C, bacteria were washed with PBS and Igs were detected using goat anti-human IgG and goat anti-human IgM both conjugated to Alexa Fluor 488 by flow cytometry analysis. In additional experiments, increasing concentration of NHS (2.5%, 10% and 30%) were used, including the two gram-negative strains *M. catarrhalis* RH4 and *H. influenzae* 3655 as controls.

### Complement-Mediated Bactericidal Activity Assay

Bacterial cells were harvested, washed in PBS, and diluted in GVB^++^. Approximately 2×10^5^ CFU/mL of bacteria were mixed with 30% NHS or 30% NHS with 50 µg/mL OmCI protein acting as a control for complement-mediated killing. At 0 h of incubation and after 1 h of incubation, 25 µL of the reaction mixtures were collected, diluted serially in PBS, and the dilutions were plated onto blood agar plates in triplicates to enumerate the starting bacterial titer. Blood agar plates were then incubated overnight at 37°C and formed colonies were enumerated. Survival of *Acinetobacter* isolates was calculated as a number of viable CFU/mL at t1, where CFU/mL at t0 was referred to input of bacteria. Concentration of 30% NHS was chosen on basis of initial titrations and the observations that quantity of serum complement present at the infection sites is limited and often referred to be approximately 20%-40% of the activity of NHS as compared to 50% in blood.

### Blood Killing Assay

Bacterial cells were harvested, washed in PBS, and diluted in PBS. Human blood was taken from healthy volunteers and treated with lepirudin (Refludan 50 μg/ml; Celgene). Approximately 2×10^6^ CFU/mL of bacteria (OD_600_ = 0.1) were mixed with full blood or blood with inhibitors (compstatin 20 µM, cytochalasin D 10 µM, OmCI 50 µg/mL) and incubated at 37°C with gentle end-over-end mixing for up to 3 h. At 0 h of incubation, and after 1, 2 and 3 h of incubation, 50 µL aliquots were collected, serially diluted in PBS, and plated onto agar plates in triplicates. After overnight incubation, colonies were enumerated and CFU/mL from each time point was calculated. We did not dilute whole blood to mimic as close as possible physiological conditions thus including phagocytes and 50% serum.

### Binding of Complement Inhibitors

For the binding of human Factor H and C4BP bacteria were harvested, washed in PBS, and diluted in GVB^++^ buffer to OD_600_ = 1. Bacteria were incubated for 1 h at 37°C with 30% heat-inactivated NHS. After incubation, bacteria were washed with PBS and stained with biotinylated anti-Factor H and anti-C4BP antibodies for 30 min at RT. After washing with PBS, biotinylated antibodies were detected with streptavidin conjugated to Alexa Fluor 647. Bound antibodies were measured using flow cytometry.

### Capsule Detection

Bacteria were harvested, washed in PBS, and diluted in PBS to OD_600_ = 1. For the detection of the K1 capsule serotype, bacteria were incubated with mouse a-K1 serotype IgM (MAb 13D6) or mouse control IgM antibodies for 30 min at RT. After washing with PBS, goat anti-mouse IgM (µ-chain) conjugated to Alexa Fluor 647 antibodies were added followed by flow cytometry analysis.

A few bacterial colonies were collected, mixed with drop of India ink (Royal Talens, 44257002) and spread on microscope slide to create a thin film. After air drying, slides were stained with 1% Crystal Violet (Merck, 115940) for app. 1 min. Subsequently, slides were left to air dry, and coverslips were mounted using ProLong Glass Antifade Mountant (Thermo Fisher Scientific, P36980). Images of stained bacteria were taken using Olympus IX53 inverted microscope with magnification 100x.

### Statistical Analyses

Statistical analyses were performed using GraphPad Prism 9. One-way ANOVA with Dunnett’s multiple-comparisons post-test was used to analyze results from bactericidal activity assays of *Acinetobacter* spp. isolates, considering input as control sample. One-way ANOVA with Dunnett’s multiple-comparisons post-test was used to analyze results from bactericidal activity assays and complement deposition assays on *A. baumannii* 307-0294 parental strain and capsule mutant strains, considering parental strain as a control sample. Two-way ANOVA with Dunnett’s post-test was used to analyze results from blood killing assay, considering sample with whole blood as a control. Two-way ANOVA with Dunnett’s post-test was used to analyze results from C4b deposition assays in depleted sera, considering sample with NHS as control sample. Significant differences are indicated with asterisks: *p < 0.05, **p < 0.01, ***p < 0.005, and ****p < 0.0001.

## Results

### *Acinetobacter* Isolates Are Resistant to Complement-Mediated Bactericidal Activity but Are Killed by Human Whole Blood

**Figure 1:**
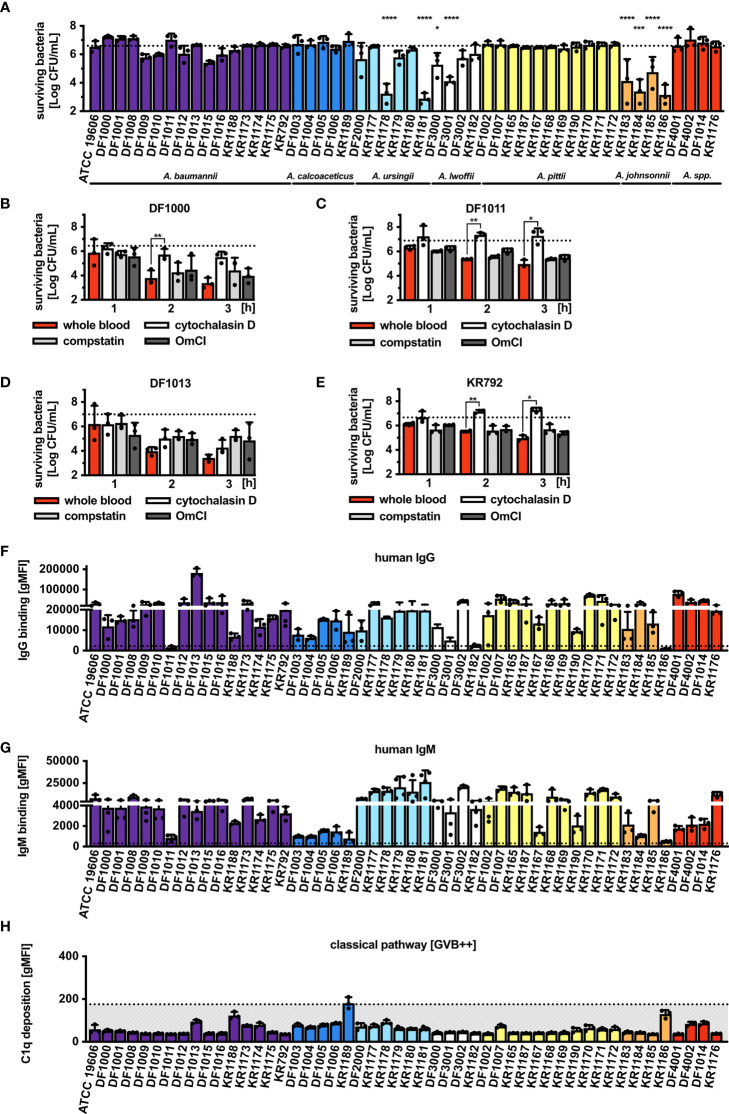
Survival of *Acinetobacter* isolates in human serum and blood, and recognition by the complement system. **(A)** Survival of *Acinetobacter* isolates in the presence of 30% NHS. Survival of isolates **(B)** *A. baumannii* DF1000, **(C)** *A. baumannii* DF1011, **(D)** *A. baumannii* DF1013 and **(E)** *A. baumannii* KR792 in human blood. Survival of bacteria was analyzed as CFU/mL, bars represent mean ± SD of at least 3 independent experiments. Horizontal dotted line refers to the starting number of bacteria used in the assay. One-way ANOVA with Dunnett’s post-test was used to analyze results from bactericidal activity assays, considering input as a control sample. Two-way ANOVA with Dunnett’s post-test was used to analyze results from blood killing assay, considering sample with full blood as a control sample. *p < 0.05, **p < 0.01, ***p < 0.005, and ****p < 0.0001. Recognition of *Acinetobacter* by human **(F)** IgG and **(G)** IgM antibodies. **(H)** Deposition of C1q on bacterial surface. **(F–H)** Bars represent gMFI ± SD of at least 3 independent experiments; horizontal dotted line represents gMFI (average + SD) measured in human IgG/IgM-depleted serum or human C1q-depleted serum acting as a binding/deposition control.

A total of 50 *Acinetobacter* strains (**Supplementary Table 1**) were assessed. Being a gram-negative bacterium, *Acinetobacter* should theoretically be directly killed by complement once MAC is deposited onto the bacterial surface resulting in lysis. To test survival of the bacteria we used pooled normal human serum (NHS). After 1 h of incubation in 30% NHS, we observed the majority of isolates survived serum challenge (**Figure 1A**) with only 8 isolates characterized by a significantly reduced but still detectable survival rate. Interestingly, none of the tested isolates was fully killed, showing a general high resistance of *Acinetobacter* to direct complement killing. Addition of C5 inhibitor (OmCI) to NHS significantly improved survival of the isolates that were otherwise partly killed by complement (**Supplementary Figure 1A**). We next tested whether *Acinetobacter* would survive in human whole blood, in which bacteria can be targeted simultaneously by the complement system and phagocytes. For these experiments we selected four isolates, which belong to the baumannii group and differ in the deposition of C4b and MAC even though they completely survive in 30% NHS. Survival of all four tested isolates was affected by human blood, especially for *Acinetobacter baumannii* DF1000 and DF1013 (**Figures 1B** **–E**). Addition of cytochalasin D, an inhibitor of phagocytosis, resulted in significantly improved survival of bacteria compared to untreated whole blood samples. Interestingly, neither compstatin, an inhibitor of C3, nor OmCI, an inhibitor of C5, fully rescued bacteria from whole blood killing. Furthermore, we observed that all 4 isolates fully survived 3 h of incubation in 50% NHS corresponding to full blood concentration (**Supplementary Figures 1B–E**). Taken together, complement alone is not sufficient to kill *Acinetobacter* and phagocytosis plays an important role in clearing bacteria during blood infection.

### *Acinetobacter* Isolates Are Recognized by Human Immunoglobulins

Since IgG and IgM activate the classical pathway of complement, we assessed whether these immunoglobulins could bind *Acinetobacter*. Incubation with 2.5% NHS showed significant binding of IgG (**Figure 1F**) and IgM (**Figure 1G**) to the majority of tested isolates. Interestingly, immunoglobulins present in the pooled NHS used in the current study also recognized the two respiratory tract pathogens *M. catarrhalis* RH4 and *H. influenzae* 3655, suggesting both specific and common recognition patterns on gram-negative bacteria (**Supplementary Figures 2A–D**). Only the three isolates *A. baumannii* DF1011, *A. lwoffi* KR1182 and *A. johnsonnii* KR1186 did not bind IgG, while IgM failed to bind to 9 isolates. Interestingly, isolates *A. baumannii* DF1011 and *A. johnsonnii* KR1186 bound neither IgG, nor IgM. Knowing that most isolates bind human immunoglobulins we tested whether the classical pathway complement component C1q was deposited on the bacterial surface. Despite several repetitions under varying conditions of time and serum concentration we could not detect significant concentrations of deposited C1q (**Figure 1H**). This could be due to the very transient nature of C1q binding to the surface of *Acinetobacter*. Recently, it was observed that while the IgG2 subclass of human antibodies fully activates classical complement pathway, very little of deposited C1q can be detected on the bacterial surface after several steps of washing (47). In support of this hypothesis is the fact that almost all isolates used in this study bound significant concentrations of human IgG2 from pooled serum (data not shown).

### The Complement System Is Activated on the Surface of *Acinetobacter* Isolates

**Figure 2:**
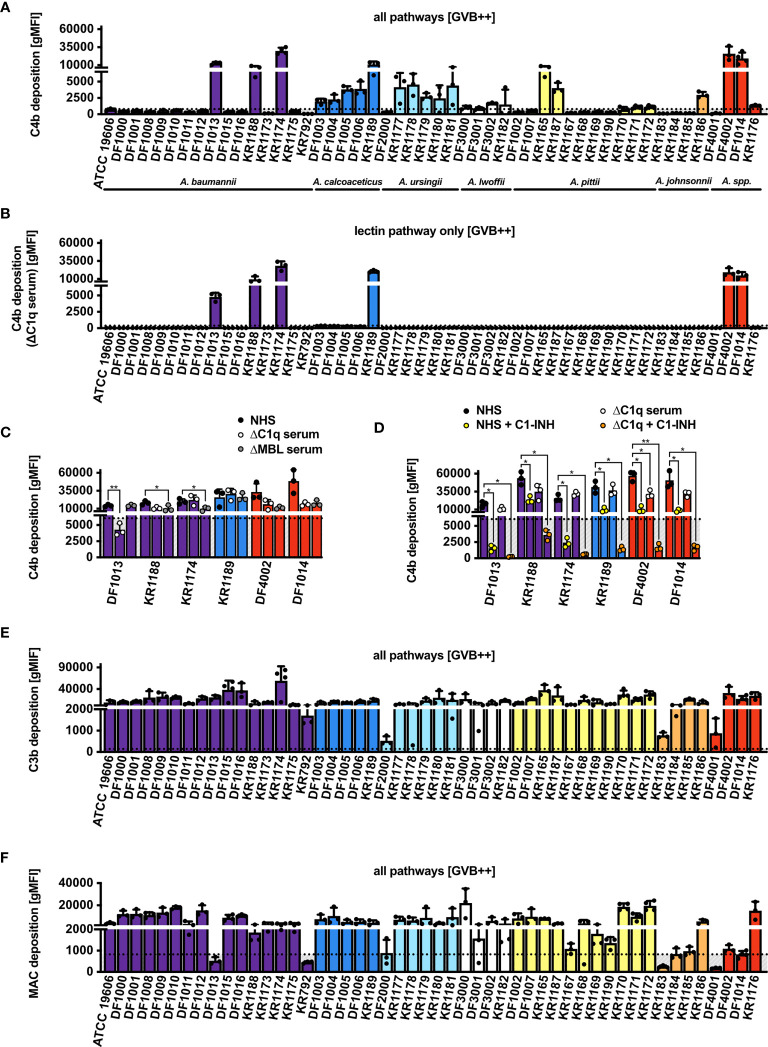
Complement deposition on *Acinetobacter* isolates *via* all pathways (GVB^++^ buffer). **(A)** Deposition of C4b on the bacterial surface in NHS. **(B)** Deposition of C4b on bacterial surface in human C1q-depleted serum. **(C)** Deposition of C4b on bacterial surface in NHS, human C1q-depleted serum, and human MBL-depleted serum. **(D)** Deposition of C4b on bacterial surface in the presence of C1-INH. Two-way ANOVA with Dunnett’s post-test was used to analyze results from C4b deposition assays in depleted sera, considering sample with NHS as a control sample. *p < 0.05, **p < 0.01. Deposition of **(E)** C3b and **(F)** MAC on bacterial surface in NHS. Bars represent gMFI ± SD of at least 3 independent experiments; horizontal dotted line represents gMFI (average + SD) measured in human sera with EDTA acting as a deposition control.

The next step was to study deposition of the sequential complement components: C4b, C3b and MAC. After incubation with 10% NHS, we observed an increased C4b deposition in approximately 40% (20 out of 50) of the isolates tested when all complement pathways were activated (**Figure 2A**). To assess whether deposition of C4b was mediated by the C1 complex, we used C1q-depleted serum and found that only 6 isolates still deposited C4b, indicating that C4d originated from the lectin pathway in this set of strains (**Figure 2B**). The remaining 14 isolates, that did not display C4b deposition in C1q-depleted serum, were opsonized with C4b likely through the C1 complex even though we could not detect C1q binding using flow cytometry. The possibility that C1q was not detected because of low binding of IgG at chosen NHS concentrations was excluded, since no C1q deposition was observed even when increasing concentrations of NHS were added, while gram-negative control strains clearly bound C1q (**Supplementary Figures 2C, D**). Furthermore, we measured C4b deposition in NHS, in addition C1q- and MBL-depleted sera, using the 6 isolates which activated the lectin pathway in the absence (**Figure 2C**) and presence of C1-INH, which inhibits serine proteases C1s, C1r associated with C1q complex, and MASPs associated with MBL (**Figure 2D**). For the isolate *A. baumannii* DF1013, we found significantly decreased C4b deposition in C1q-depleted serum, while isolates *A. baumannii* KR1188 and *A. baumannii* KR1174 deposited significantly less C4b in MBL-depleted serum. We observed a similar deposition of C4b from all used sera for *A. calcoaceticus* KR1189, which might indicate that deposition was mediated *via* both C1q and MBL or *via* other less well characterized proteins, for example ficolins. C1-INH added to the C1q-deficient serum strongly inhibited C4b deposition on all 6 isolates. Of note, C1q, MBL and ficolin associated proteases are sensitive to C1-INH. Next, we observed a strong deposition of C3b on most isolates, with only *A. ursingii* DF2000, *A. pittii* KR1183 and *A. johnsonnii* DF4001 isolates depositing clearly less C3b (**Figure 2E**). Finally, we tested MAC deposition, the final step of the complement cascade, and detected MAC on the surface of most isolates except for 11, which deposited evidently less MAC than other isolates, or not at all (**Figure 2F**).

### *Acinetobacter* Isolates Trigger the Alternative Pathway of Complement

**Figure 3:**
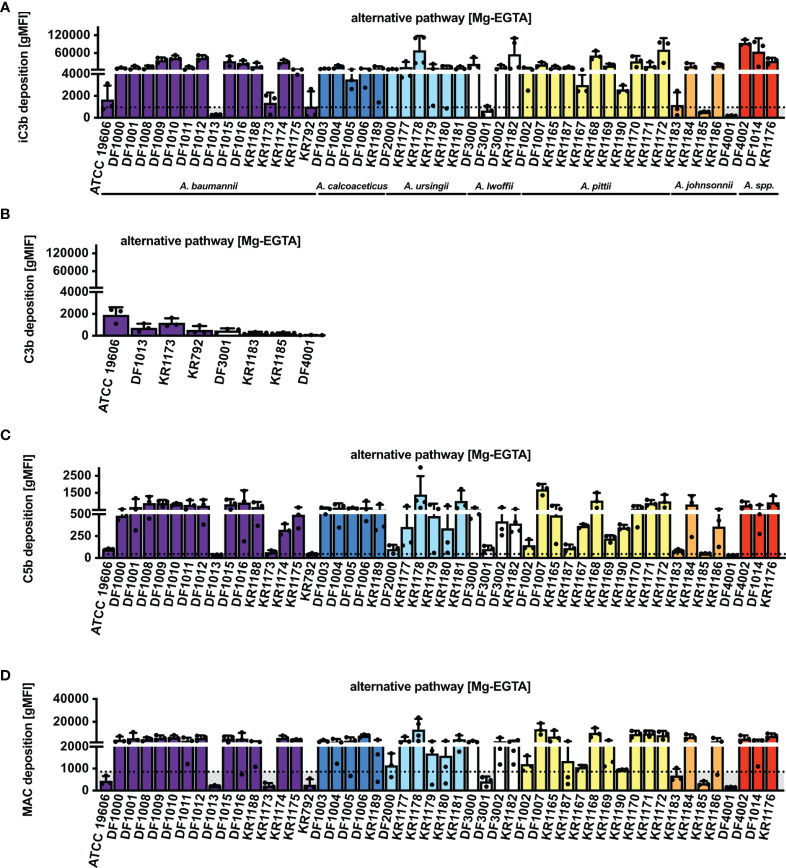
Complement deposition on *Acinetobacter* isolates *via* alternative pathway (Mg-EGTA buffer). Deposition of **(A)** iC3b, **(B)** C3b, **(C)** C5b and **(D)** MAC on bacterial surface in NHS. Bars represent gMFI ± SD of at least 3 independent experiments; horizontal dotted line represents gMFI (average + SD) measured in NHS with EDTA acting as a deposition control.

Previously described experiments were performed in a buffer containing calcium and magnesium, thus allowing activation of all complement pathways, in particular the classical and the lectin pathways. Using Mg-EGTA buffer, which allows study of the alternative pathway activation only, we observed deposition of iC3b on 84% of isolates (42/50) (**Figure 3A**). The 8 isolates for which iC3b deposition was not observed were additionally tested for intact C3b deposition. Similarly, all 8 isolates deposited little C3b, equivalent to the amount of iC3b (**Figure 3B**). Furthermore, C5b deposition was detected on 78% of isolates (39/50) (**Figure 3C**). Accordingly, MAC deposition was not observed on the 11 isolates that lacked C5b deposition (**Figure 3D**).

### The Capsule of *Acinetobacter* spp. Modulates Complement Activation

**Figure 4:**
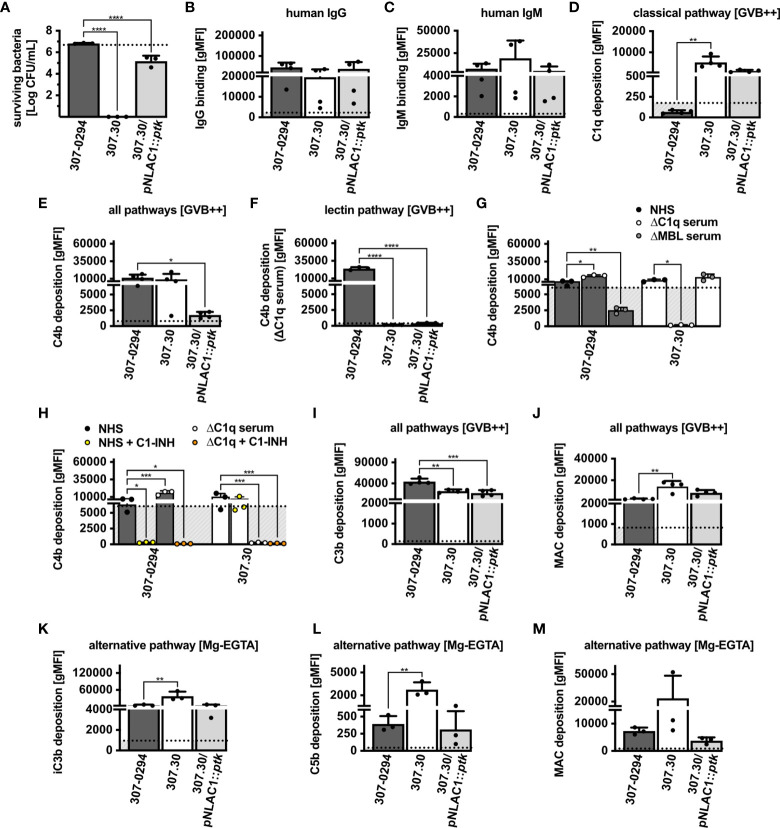
Survival and complement system activation on *A. baumannii* mutant strains. **(A)** Survival of wt and capsule mutant strains in 30% NHS. Binding of human **(B)** IgG and **(C)** IgM. **(D)** Deposition of C1q. Deposition of C4b in **(E)** NHS, **(F)** human C1q-depleted serum, **(G)** NHS, human C1-depleted serum and MBL-depleted serum, **(H)** the presence of C1-INH. Deposition *via* all pathways of **(I)** C3b and **(J)** MAC and deposition *via* alternative pathway of **(K)** iC3b, **(L)** C5b and **(M)** MAC. **(B–M)** Bars represent gMFI ± SD of at least 3 independent experiments; horizontal dotted line represents gMFI (average + SD) measured in human sera with EDTA acting as a deposition control. One-way ANOVA with Dunnett’s post-test was used to analyze results from bactericidal activity assays and complement deposition assays, considering the parental AB307-0294 wt strain as control sample. Two-way ANOVA with Dunnett’s post-test was used to analyze results from C4b deposition assays in depleted sera, considering sample with NHS as a control sample. *p < 0.05, **p < 0.01, ***p < 0.005, and ****p < 0.0001.

*Acinetobacter* spp. produce a polysaccharide capsule for protection from the environment, which in turn could also mitigate complement-mediated bactericidal activity (15). To study the importance of the capsule on serum survival, complement recognition and activation we used a parental *A. baumannii* wt strain (AB307-0294), an isogenic mutant derivative that was capsule deficient (AB307.30), and a strain in which capsule production was restored *via* complementation (AB307.30/pNLAC1::*ptk*) (**Supplementary Table 2**) (16). We observed that the AB307-0294 wt strain survived in serum, whereas the isogenic capsule negative mutant derivative AB307.30 was completely killed (**Figure 4A**). In the same experiment, the capsule complemented strain AB307.30/pNLAC1::*ptk* survived in serum. Its survival rate was, however, significantly reduced compared to the parental AB307-0294 strain, suggesting that the capsule was not fully restored to the wt levels. After addition of OmCI, all strains fully survived treatment in NHS (**Supplementary Figure 1F**), indicating a complement-mediated killing in the capsule negative mutant derivative. *A. baumannii* wild-type and mutant strains were recognized by human immunoglobulins IgG (**Figure 4B**) and IgM at similar levels (**Figure 4C**). No C1q deposition was detected on the wt but both mutants showed significant deposition of C1q on the surface (**Figure 4D**). We observed significant C4b deposition from NHS on the wt and capsule negative mutant derivative, while the capsule complemented AB307.30/pNLAC1::*ptk* deposited little C4b (**Figure 4E**). Interestingly, only the wt strain deposited C4b from C1q-depleted serum (**Figure 4F**). Absence of C4b deposition in MBL-depleted serum indicated that wt strain activated lectin pathway (**Figure 4G**). By contrast, both capsule mutants deposited C4b in MBL but not C1q-depleted serum, demonstrating activation of the classical pathway. C1-INH clearly inhibited complement deposition on the wt strain in both NHS and C1-depleted serum, further confirming activation of the lectin pathway on this strain. C1-INH failed to block C4b deposition on the mutant strain, possibly due to the lower efficacy of inhibition of C1 complex in comparison to MBL complex (**Figure 4H**). All strains showed both C3b (**Figure 4I**) and MAC deposition (**Figure 4J**) in a buffer allowing classical and lectin pathways. Furthermore, we observed deposition of iC3b *via* the alternative pathway (**Figure 4K**). Intriguingly, *A. baumannii* wt and AB307.30/pNLAC1::*ptk* capsulated strains, which survived in human serum, showed a tendency towards less C5b (**Figure 4L**) and MAC (**Figure 4M**) deposited on their surfaces, compared to the capsule negative mutant strain. Taken together, our data indicate that the capsule is protecting bacteria from MAC-mediated lysis.

### *Acinetobacter* spp. Polysaccharide Capsule Thickness Varies Between Isolates

**Figure 5:**
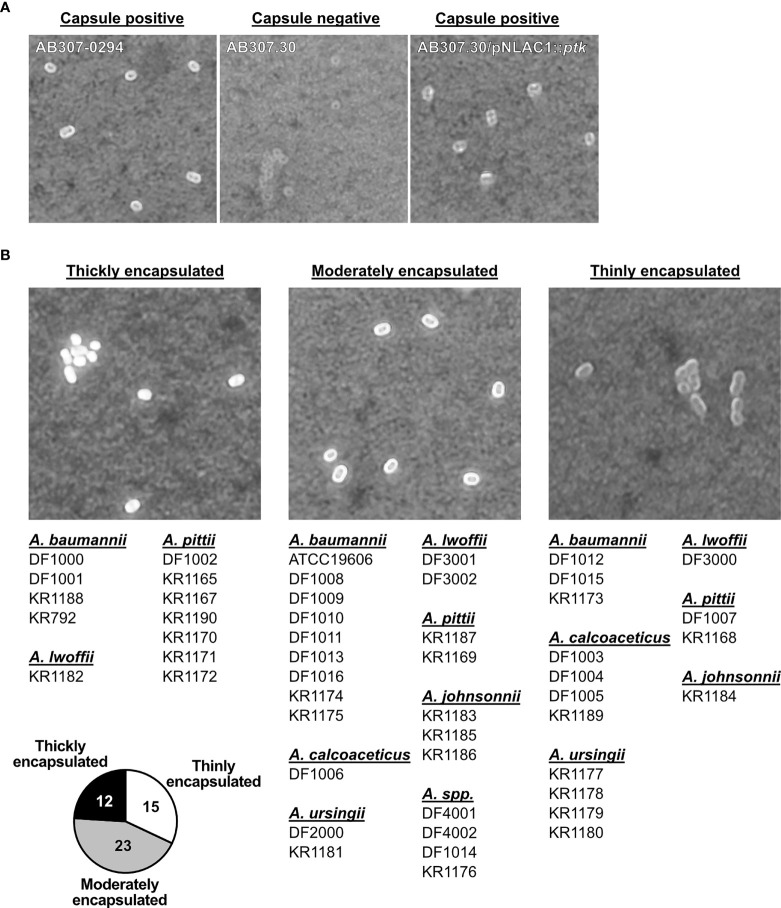
Detection of capsule on *Acinetobacter* isolates. **(A)** Detection of the capsule on *A. baumannii* wt and capsule mutant strains, and **(B)** *Acinetobacter* spp. isolates, using India ink and crystal violet staining. Brightfield microscopy of bacterial cell with capsule (white halo surrounding cell). Representative image of each strain is presented. Isolates are grouped into 3 groups (thickly encapsulated, moderately encapsulated, and thinly encapsulated), corresponding to the bacterial encapsulation level. Pie chart represents number of isolates in each group.

The majority of *Acinetobacter* spp. are encapsulated (15, 17, 18), and since we found significant differences in complement deposition on various clinical isolates, we wanted to determine whether there was a difference in capsule thickness in our strain collection. We performed India ink staining in conjunction with crystal violet to identify the presence of capsule. Encapsulated isolates are expected to be surrounded by a white halo (48, 49). To validate the assay, we demonstrated that India ink staining was positive for *A. baumannii* AB307-0294 (wt, capsule positive) as well as AB307.30/pNLAC1::*ptk* (complemented mutant derivative, capsule positive), but negative for AB307.30 (capsule negative) (**Figure 5A**). This was further confirmed using the mouse α-K1 capsule antibody (MAb 13D6) (16) (**Supplementary Figure 3A**). Next, we assessed our library of 50 isolates and based on the India Ink stain results were grouped according to their capsule size into thickly, moderately, and thinly encapsulated (**Figure 5B**). Representative images of stained capsule for each clinical *Acinetobacter* isolate are presented in **Supplementary Figures 3B–H**. Twelve isolates (24%) appeared to be thickly encapsulated, 23 isolates (46%) appeared to be moderately encapsulated, while 15 isolates (30%) were thinly encapsulated. Among the 50 clinical strains, the α-K1 capsule antibody only bound *A. baumannii* KR1174 isolate, indicating KR1174 possessed a K1 serotype (data not shown); the other 49 isolates presumably expressed a different capsule type. Interestingly, we could not detect any non-capsulated strains, indicating that a capsular exopolysaccharide, albeit with quantitative variation, is a ubiquitous phenotype among clinical *Acinetobacter* isolates.

## Discussion

Bacteria from the genus *Acinetobacter* are emerging among the most important human pathogens. While being relatively harmless for healthy people, hospital-related infections for immunocompromised hosts are often fatal (1–5). Particularly worrying is multi- and extensive drug resistance spreading among *Acinetobacter* species (10). Although many virulence factors and escape mechanisms from host defense factors for *Acinetobacter* are already known, there is a limited body of knowledge on how *A. baumannii* avoids complement-mediated killing. Therefore, in this study we aimed to further investigate recognition and activation of the complement system on *Acinetobacter* subspecies in general, and thus identify potential escape mechanisms.

One of the most important effector functions of complement is assembly of the MAC and subsequent direct lysis of gram-negative bacteria such as *Acinetobacter*. We found, that although most of isolates deposited significant amount of MAC, most of them fully survived treatment in 30% NHS, and some isolates survived even in 50% NHS. Similar serum-resistance of *Acinetobacter* species was documented previously (50, 51). Interestingly, we identified a few isolates that were partially killed by NHS, and, for some of them, addition of the C5 inhibitor OmCI fully rescued their survival, indicating that serum sensitivity was mediated by complement activation. However, three strains (*A. ursingii* KR1178, *A. ursingii* KR1181 and *A. johnsonii* KR1186) remained sensitive to serum even in the presence of OmCI. This suggests that other factors, such as antimicrobial peptides like LL-37 (52) or lysozyme, might be involved in the killing of *Acinetobacter*, as previously documented (35). To escape complement-mediated lysis pathogens bind complement inhibitors, mainly FH and C4BP. Similar mechanism was suggested for one strain of *A. baumannii* (ATCC 19606), where outer membrane porin OmpA, bound FH, contributing to the serum resistance (34). Interestingly, another report excluded the possibility of an active binding of FH by *A. baumannii* clinical isolates (50). Using several approaches including NHS and purified proteins, we could not detect significant binding of FH or C4BP for any isolate tested in our study (**Supplementary Figures 2E, F**). Given the previous reports and our observations, we can speculate that binding of FH and C4BP inhibitors is not a common mechanism among *Acinetobacter* spp. In our experimental setup, two isolates tested, *A. ursingii* DF2000 and *A. lwoffii* DF3002, bound more FH than other isolates. The measured signal was still, however, too close to background levels to be considered positive in a statistically significant manner. This might, nevertheless, indicate that other *Acinetobacter* subspecies than *A. baumannii* may be able to bind complement inhibitors. Further studies are, however, required to obtain certain proof on this matter.

We have established that the production of a capsule is one mechanism that mediates resistance to complement-mediated bactericidal activity by decreasing MAC deposition and lysis. *Acinetobacter* species are coated with a polysaccharide layer, which protects cells from direct lysis by complement or antimicrobial compounds, and from phagocytic killing (15–18, 53). Using a staining method with India ink and crystal violet we observed that all isolates were encapsulated to a varying degree. Interestingly, isolates that were affected by complement-mediated killing in NHS were also capsulated. Earlier studies have shown that *Acinetobacter* species differ greatly in capsule composition, since the K gene locus encodes for around hundred different capsule types (18, 54). The capsule is considered as one of the targets for potential anti-*bacterial* treatment and promising results were shown in experiments with passive immunization in mice using monoclonal antibodies directed against *A. baumannii* (55, 56). However, abundance of many different serotypes makes development of general therapy more difficult, since monoclonal antibodies would be predicted to be either capsule type specific or perhaps specific to a few capsule types that share epitopes (55, 57). Our study shows the importance of the capsule as one of the significant immune evasion mechanisms of *Acinetobacter* spp. Using *A. baumannii* AB307-2094 wt strain and an isogenic capsule deficient mutant derivative we showed similar recognition and activation of complement on the wt and mutant strain, yet capsule negative bacteria were completely killed in serum. This phenotype was largely but not fully reversed in *A. baumannii* 307.30/pNLAC1::*ptk* mutant strain genetically complemented to restore capsule expression. The *ptk* gene is involved in the synthesis of capsule in bacteria (58), thus it is possible that re-expressed protein in the mutant strain was not fully functional as in the parental AB307-0294 wt strain. Nonetheless, India ink staining showed expression of the capsule in capsule complemented mutant, and in consequence resistance to NHS was restored.

Other research groups attempted to clarify the molecular mechanisms underlying serum resistance in *Acinetobacter*. Those observations appear, however, to be inconsistent or limited to few isolates (59, 60). Such discrepant observations highlight the importance of our study, as well as following studies concerning *Acinetobacter* escape mechanisms.

**Figure 6:**
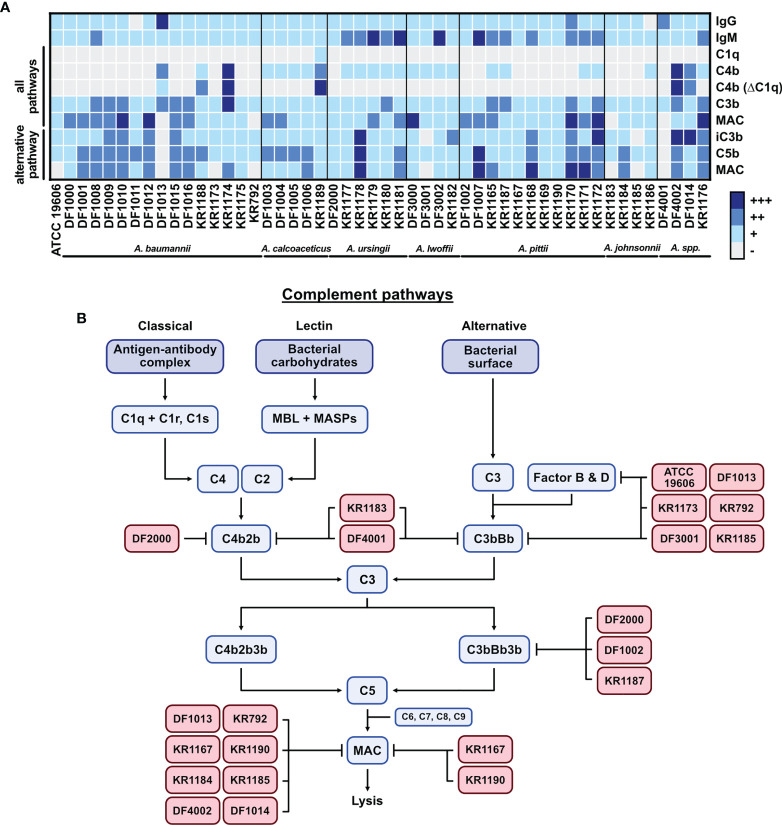
Graphical summary of the study. **(A)** Summary of the complement activation data presented as a heat-map of deposition of each tested protein. Flow cytometry results for each isolate are arranged into 4 groups (+++ highest deposition, ++ moderate deposition, + low deposition, - no deposition/background level). Groups are created based on the percentage of the highest gMFI value for each deposited component. **(B)** The complement system activation cascade. *Acinetobacter* isolates with their potential targets in the cascade are presented.

In this study, we screened step-by-step activation of complement pathways on clinical isolates of *Acinetobacter* (**Figure 6A**). *Acinetobacter* isolates were generally recognized by human IgGs and IgMs in NHS. NHS used in this study was a pool obtained from 12 healthy volunteers, which most likely contained antibodies recognizing common bacterial antigens such as LPS shared by gram-negative pathogens. In support, immunoglobulins were deposited from the same NHS on *M. catarrhalis* RH4 and *H. influenzae* 3655 and could be recognizing both specific and common antigenic patterns from gram-negative bacteria. Furthermore, we cannot exclude that some of the donors have been previously colonized or infected by *A. baumannii*. *Acinetobacter* species are abundant in the environment; thus, we are constantly in contact with non-pathogenic strains (61, 62). This abundance of both adaptive (acquired), cross-reactive and innate IgM antibody binding *Acinetobacter* may, in part, explain why these bacteria pose no threat for healthy people (63, 64). A weakness of this study was that we did not determine the exact nature of anti-*Acinetobacter* antibodies but that was not the initial goal of the study. This will, however, be the impetus for future studies.

Moreover, we could not detect any binding of C1q to isolates with deposited C4b although such deposition was abolished in C1q-depleted serum, yet C1q and C4b deposition was clearly detected on two other gram-negative control bacterial strains. We hypothesize that bacteria bind C1q transiently, as previously observed for bacteria coated with IgG2 (47). On the other hand, we detected a clear binding of C1q to all capsule mutant strains. This indicates that the capsule is hindering strong binding of C1q on the bacterial surface. Interestingly, not all isolates had deposited C4b, and observed deposition of C4b could be mediated by C1 complex (classical pathway) or MBL complex (lectin pathway). C4b deposition was observed on mutant strains, where *A. baumannii* AB307-0294 wt strain activated lectin pathway, while isogenic capsule deficient mutant derivatives activated the classical pathway. These results indicate a switch in complement recognition depending on the presence or absence of the protective capsule.

Moreover, while most isolates deposited C3b/iC3b, several isolates deposited significantly less MAC, indicating virulence mechanisms protecting from MAC and direct lysis. Furthermore, several isolates did not carry any deposited C3b *via* the alternative pathway and as a consequence, these isolates deposited much less C5b and MAC compared to other isolates. This suggests that *A. baumannii* possesses an undefined escape mechanism directed against the alternative pathway of complement activation.

Complement activation triggers a cascade of proteins by the classical, lectin or alternative pathways (20). Activation of pathways leads to the formation of C3 and C5 convertases, which cleave and activate C3 and C5. The final step of the cascade consists in MAC deposition and insertion into the bacterial cell outer membrane, causing direct lysis of gram-negative bacteria. **Figure 6B** presents a graphical summary with the potential complement targets of some serum resistant *Acinetobacter* isolates. Isolates *A. baumannii* KR1183 and *Acinetobacter* spp. DF4001 inhibit both classical pathway and alternative pathway convertases since neither C3b/iC3b nor MAC are deposited on their surface in all buffer conditions tested. Isolates *A. baumannii* ATCC 19606, *A. baumannii* DF1013, *A. baumannii* KR1173, *A. baumannii* KR792, *A. lwoffii* DF3001 and *A. johnsonnii* KR1185 selectively block the alternative pathway convertase, either at C3 or Factor B/Factor D levels, without affecting C3b deposition through the classical pathway. Interestingly, isolates *A. johnsonnii* KR1183 and KR1185 are killed by complement even though they have only limited MAC deposition on their surface. Similarly, isolate *A. lwoffii* DF3001 completely blocked alternative pathway, however its survival in NHS was significantly reduced. Of note, neither C3b nor MAC were recovered on the surface of isolate *A. ursingii* DF2000 *via* any pathway, however, this isolate had high levels of deposited iC3b suggesting a possible mechanism in promoting cleavage of C3. Isolates *A. pittii* DF1002 and KR1187 inhibited alternative pathway at the C5 level. MAC deposition at the C9 level was hampered by several isolates (*A. baumannii* DF1013, *A. baumannii* KR792, *A. pittii* KR1167, *A. pittii* KR1190, *A. johnsonnii* KR1184, *A. johnsonnii* KR1185, *Acinetobacter* spp. DF4002, *Acinetobacter *spp. DF1014) in the classical/lectin pathway activation, thus targeting either C5 convertase (C4b2b3b), or MAC directly. Interestingly, isolates *A. pittii* KR1167 and *A. pittii* KR1190 blocked MAC formation in all pathways, even though C3b was deposited on the surface. This indicates the existence of a mechanism protecting bacteria from MAC deposition. Targeting terminal pathway of the cascade does not protect bacteria from C3b opsonization, i.e., *A. baumannii* KR792 and *A. baumannii* KR1013, which were affected by phagocytosis in whole blood, but protects from formation of the MAC and direct lysis. In contrast, the alternative pathway seems to be blocked already while forming C3 convertase (C3bBb). This in consequence blocks the whole activation cascade, preventing deposition of C5b and finally MAC. The alternative C3 convertase could be inhibited by a direct disruption of the convertase, or by blocking Factor D, or Factor B. Of note, some AMR strains, i.e., KR1175, KR1179, KR1180 and KR1181, do not inhibit the complement cascade. However, some other AMR strains (i.e., KR1173, KR792 and KR1167) hamper the complement cascade at various levels. These observations further indicate that *Acinetobacter* strains developed different mechanisms to survive in the host.

Using four serum-resistant isolates (DF1000, DF1011, DF1013, KR792) we observed partial killing after incubation in human whole blood. Complement-mediated killing seemed to be ineffective for those isolates since the addition of complement inhibitors did not protect bacteria from killing, while the presence of cytochalasin D, a phagocytosis inhibitor, significantly rescued bacteria. This strongly suggests that phagocytosis carried out by human cells in whole blood was a crucial mechanism involved in elimination of bacteria. Indeed, that was showed in previous studies, where neutrophils and tissue macrophages contributed to the killing of *Acinetobacter* cells (24, 25). We observed that most of the isolates bound significant amount of Igs, which directly could activate phagocytic cells *via* Fc receptors (22, 23). Furthermore, we observed significant opsonization of bacteria with iC3b, a main ligand of phagocytic receptor CR3, which should target bacteria for phagocytosis, while release of anaphylatoxins C3a and C5a could attract and activate immune cells (20). A better understanding of *Acinetobacter* escape mechanism(s) is, however, required to fully delineate pathogenesis.

We demonstrated that, although successfully recognized by complement, the majority of the *Acinetobacter* clinical isolates tested in this study were serum resistant. Few previous studies have described *Acinetobacter* virulence factors, while mechanisms that block the complement cascade are still unknown. Our screening highlighted some isolates which can block complement activation at different levels, suggesting the existence of multiple novel virulence factors that contribute to resistance to complement-mediated bactericidal activity in *A. baumannii*. A better understanding of escape mechanisms in *Acinetobacter* would lend insight into the development of novel treatment options not based exclusively on antibiotics.

## Conclusion

In conclusion, our data showed that *Acinetobacter* clinical isolates were recognized by immunoglobulins from pooled human serum, and the complement was efficiently activated. Despite complement activation, pathogens survived in human serum, showing high resistance to complement-mediated killing. We also demonstrated that the production of a capsular polysaccharide is one mechanism that mediates resistance to complement-mediated bactericidal activity by preventing MAC deposition and lysis.

## Data Availability Statement

The original contributions presented in the study are included in the article/**Supplementary Material**. Further inquiries can be directed to the corresponding author.

## Ethics Statement

The studies involving human participants were reviewed and approved by Ethics committee in Lund, Sweden (permit 2019/14). The patients/participants provided their written informed consent to participate in this study.

## Author Contributions

MM, SB, and AB designed the study and analyzed experimental data together with KR. MM and SB conducted experiments. ML, DF, and KR provided clinical isolates of *Acinetobacter* bacteria. TR provided wild-type strain and mutant strains of *A. baumannii*. MM, SB, and AB wrote the first draft of the manuscript. All authors contributed to the final draft of the manuscript and approved the submitted version.

## Funding

The study was supported by grants from the European Union MSCA project CORVOS 860044 (supporting doctoral education of MM); Swedish Research Council (2018-02392 to AB, and 2019-01053 to KR), the Österlund Foundation (to AB and to KR), the Torsten Söberberg Foundation and grant for clinical research (ALF) (to AB and to KR); grants from Sten K. Johnsons Foundation (2019), the Tore Nilsson’s Foundation (2019-00750 and 2020-00832), the Royal Physiographic Society of Lund (40824 and 41407), the O. E. och Edla Johanssons Foundation, The Lars Hierta Memorial Foundation (FO2020-0257), Längmanska kulturfonden (BA20-1272 and BA21-0550) and Clas Groschinskys Fondation (M21106) (to SB); the Department of Veterans Affairs VA Merit Review (1I01BX004677-01A1) (to TR); the Anna and Edwin Berger Foundation, Swedish Heart Lung Foundation (20180401) and the Skåne County Council’s research and development foundation (to KR).

## Conflict of Interest

The authors declare that the research was conducted in the absence of any commercial or financial relationships that could be construed as a potential conflict of interest.

## Publisher’s Note

All claims expressed in this article are solely those of the authors and do not necessarily represent those of their affiliated organizations, or those of the publisher, the editors and the reviewers. Any product that may be evaluated in this article, or claim that may be made by its manufacturer, is not guaranteed or endorsed by the publisher.

## Supplementary Material

Supplementary Figure 1 Survival of *Acinetobacter* isolates in human serum. **(A)** Survival of *Acinetobacter* isolates in NHS in the presence of OmCI inhibitor. Survival of **(B)** *A. baumannii* DF1000, **(C)** *A. baumannii* DF1011, **(D)** *A. baumannii* DF1013 and **(E)** *A. baumannii* KR792 in 3 concentrations of NHS at 3 time points. **(F)** Survival of wt capsule mutant strains in NHS in the presence of OmCI inhibitor. Survival of bacteria was analyzed as CFU/mL, bars represent mean +/- SD of at least 3 independent experiments. Horizontal dotted line refers to the starting number of bacteria used in the assay. One-way ANOVA with Dunnett’s multiple-comparisons post-test was used to analyze results from bactericidal activity assays, considering input (for *Acinetobacter* spp. isolates) or *A. baumannii* 307-0294 wt (for mutant strains) as control sample. ***p* < 0.01, and *****p* < 0.0001.

Supplementary Figure 2 Complement recognition and inhibitors binding to *Acinetobacter* isolates. Recognition of two *Acinetobacter* isolates*, Moraxella catarrhalis* RH4 *and Haemophilus influenzae* 3655 by human **(A)** IgG and **(B)** IgM antibodies. Deposition of **(C)** C1q and **(D)** C4b on the bacterial surface. Bars represent gMFI ± SD of at least 3 independent experiments; horizontal dotted line represents gMFI (average + SD) measured in depleted sera or NHS with EDTA acting as a deposition control. Binding of complement inhibitors **(E)** Factor H and **(F)** C4BP. Bars represent gMFI +/- SD of at least 3 independent experiments; horizontal dotted line represents gMFI (average + SD) measured from unspecific antibody signal acting as a binding control.

Supplementary Figure 3 Detection of the capsule on *Acinetobacter* isolates. **(A)** Detection of K1 serotype capsule using the specific α-capsule antibody MAb 13D6 on *A. baumannii* AB307-0294 parental strain (capsule positive) and isogenic mutant derivatives AB307.30 (capsule negative), AB307.30/pNLAC1::*ptk* (capsule positive). Bars represent gMFI ± SD of at least 3 independent experiments; horizontal dotted line represents gMFI (average + SD) measured for IgM isotype control antibody acting as a binding control **(B–H)** Detection of the capsule on bacteria using India ink and crystal violet staining. Brightfield microscopy of bacterial cell with capsule (white halo surrounding cell). Representative image of each *Acinetobacter* isolates is presented: **(B)** *A. baumannii* isolates, **(C)** *A. calcoaceticus* isolates **(D)** *A. ursingii* isolates, **(E)** *A. lwoffii* isolates, **(F)** *A. pittii* isolates, **(G)** *A. johnsonnii* isolates, **(H)** A. spp isolates.

## Abbreviations

AMR, antimicrobial resistance; C4BP, C4-binding protein; CFU, colony forming unit; C1-INH, C1-inhibitor; FH, Factor H; gMFI, geometric mean fluorescence intensity; GVB, gelatin veronal buffer; ICU, intensive care unit; Igs, immunoglobulins; mAb, monoclonal antibody; MAC, membrane attack complex; MBL, mannose-binding lectin; MDR, multidrug resistance; NETs, neutrophil extracellular traps; NHS, normal human serum; OD, optical density; OmCI, *Ornithodoros moubata* complement inhibitor; pAb, polyclonal antibody; ROS, reactive oxygen species; wt, wild-type; XDR, extensive drug resistance.
