# Supplementary figures and images for: Clinical Isolates of Acinetobacter spp. Are Highly Serum Resistant Despite Efficient Recognition by the Complement System
Source: Front Immunol. 2022 Jan 31;13:814193. doi: 10.3389/fimmu.2022.814193 (PMC8841485; doi:10.3389/fimmu.2022.814193)

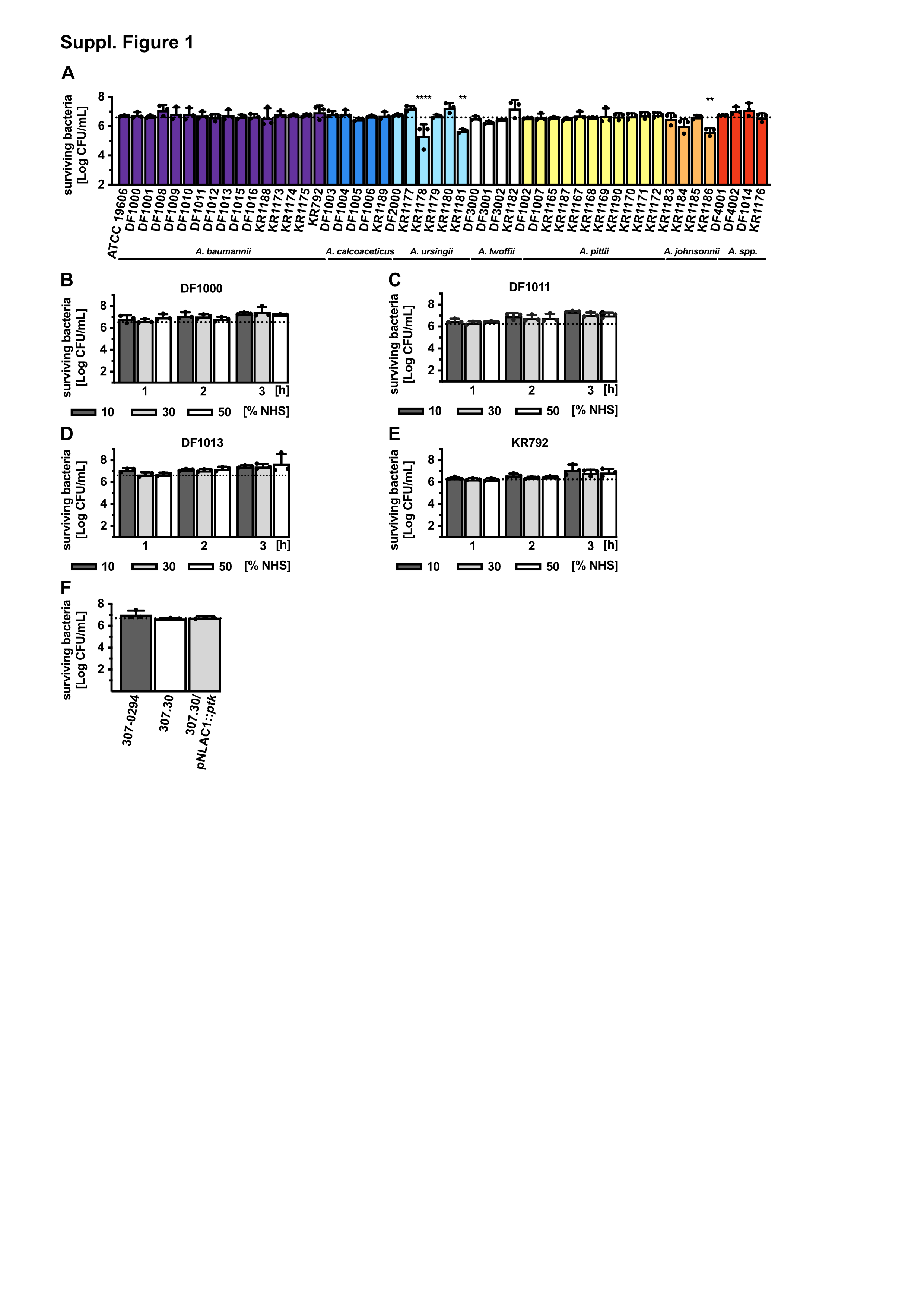

Supplement: Supplementary Figure 1 — Survival of Acinetobacter isolates in human serum. (A) Survival of Acinetobacter isolates in NHS in the presence of OmCI inhibitor. Survival of (B) A. baumannii DF1000, (C) A. baumannii DF1011, (D) A. baumannii DF1013 and (E) A. baumannii KR792 in 3 concentrations of NHS at 3 time points. (F) Survival of wt capsule mutant strains in NHS in the presence of OmCI inhibitor. Survival of bacteria was analyzed as CFU/mL, bars represent mean +/- SD of at least 3 independent experiments. Horizontal dotted line refers to the starting number of bacteria used in the assay. One-way ANOVA with Dunnett’s multiple-comparisons post-test was used to analyze results from bactericidal activity assays, considering input (for Acinetobacter spp. isolates) or A. baumannii 307-0294 wt (for mutant strains) as control sample. **p < 0.01, and ****p < 0.0001. [file Image_1.tiff]

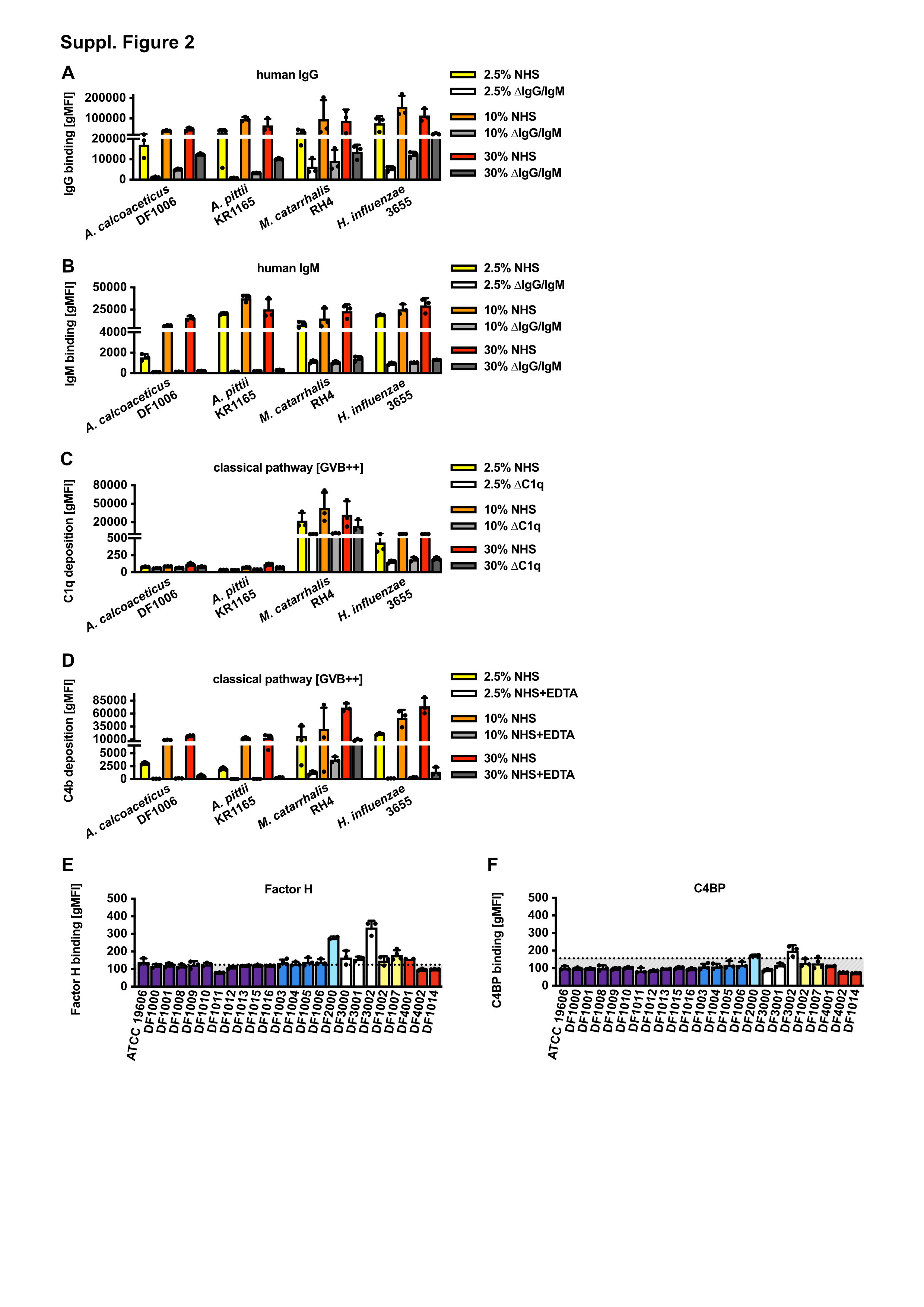

Supplement: Supplementary Figure 2 — Complement recognition and inhibitors binding to Acinetobacter isolates. Recognition of two Acinetobacter isolates, Moraxella catarrhalis RH4 and Haemophilus influenzae 3655 by human (A) IgG and (B) IgM antibodies. Deposition of (C) C1q and (D) C4b on the bacterial surface. Bars represent gMFI ± SD of at least 3 independent experiments; horizontal dotted line represents gMFI (average + SD) measured in depleted sera or NHS with EDTA acting as a deposition control. Binding of complement inhibitors (E) Factor H and (F) C4BP. Bars represent gMFI +/- SD of at least 3 independent experiments; horizontal dotted line represents gMFI (average + SD) measured from unspecific antibody signal acting as a binding control. [file Image_2.tiff]

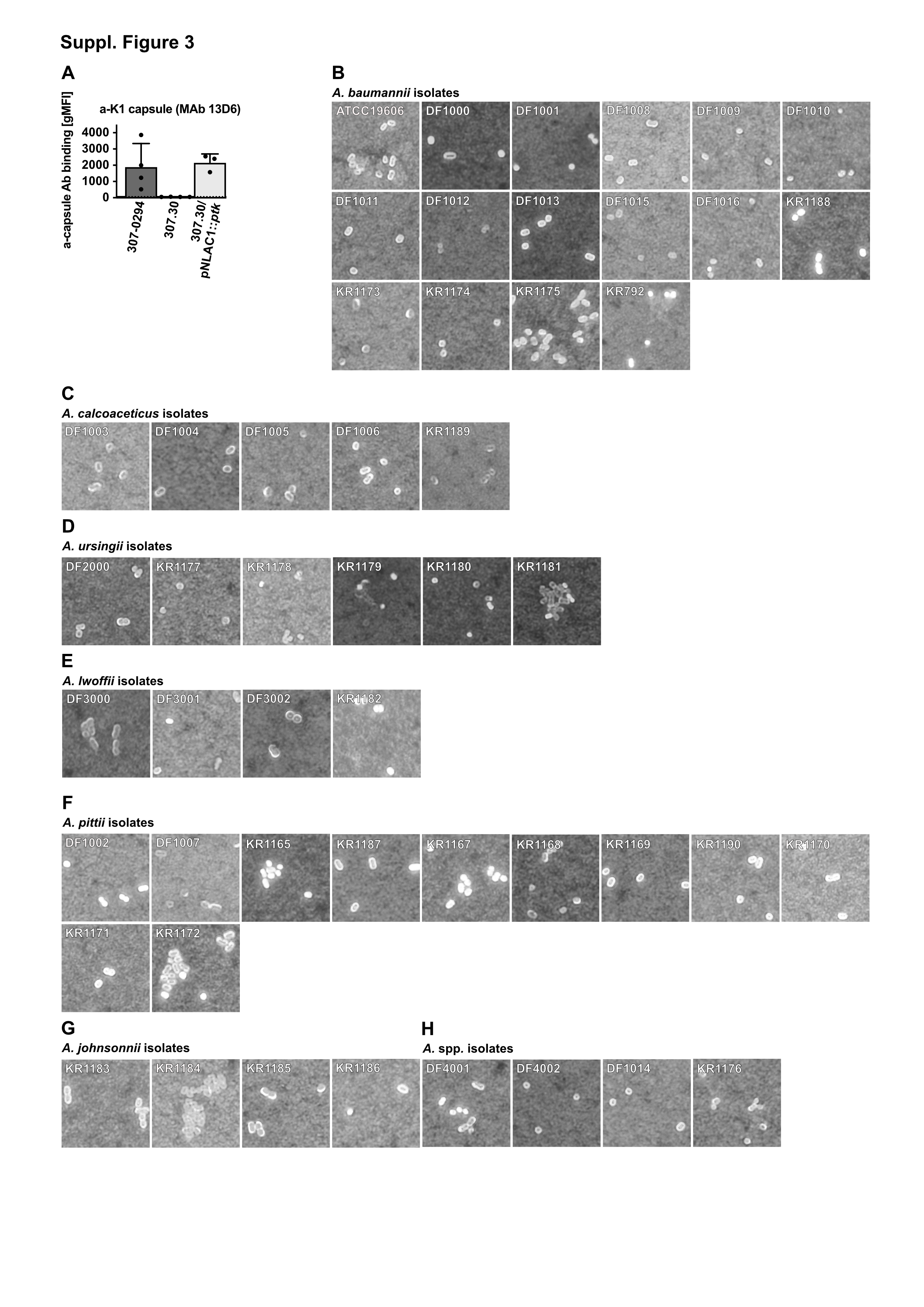

Supplement: Supplementary Figure 3 — Detection of the capsule on Acinetobacter isolates. (A) Detection of K1 serotype capsule using the specific α-capsule antibody MAb 13D6 on A. baumannii AB307-0294 parental strain (capsule positive) and isogenic mutant derivatives AB307.30 (capsule negative), AB307.30/pNLAC1::ptk (capsule positive). Bars represent gMFI ± SD of at least 3 independent experiments; horizontal dotted line represents gMFI (average + SD) measured for IgM isotype control antibody acting as a binding control (B–H) Detection of the capsule on bacteria using India ink and crystal violet staining. Brightfield microscopy of bacterial cell with capsule (white halo surrounding cell). Representative image of each Acinetobacter isolates is presented: (B) A. baumannii isolates, (C) A. calcoaceticus isolates (D) A. ursingii isolates, (E) A. lwoffii isolates, (F) A. pittii isolates, (G) A. johnsonnii isolates, (H) A. spp isolates. [file Image_3.tiff]
