# Supplementary material for: Clinical Isolates of Acinetobacter spp. Are Highly Serum Resistant Despite Efficient Recognition by the Complement System
Source: Front Immunol. 2022 Jan 31;13:814193. doi: 10.3389/fimmu.2022.814193 (PMC8841485; doi:10.3389/fimmu.2022.814193)
Supplement: Supplementary file 4 [file Table_1.docx]

**Suppl. Table 1. List of *Acinetobacter* strains used in the study.** ^a^ ATCC 19606 laboratory strain purchased from CCUG (Göteborg, Sweden), ^b^ isolates form Royal Victoria Hospital (Belfast, Northern Ireland), ^c^ isolates from Clinical Microbiology, Laboratory Medicine Skåne (Lund, Sweden); ^1, 2, 3, 4, 5, 6, 7, 8^ patients from which more than one strain was isolated.

|  | | | **ANTIBIOTICS RESISTANCE** | | |
| --- | --- | --- | --- | --- | --- |
| **NUMBER** | **STRAIN** | **SOURCE** | **SENSITIVE** | **INTERMEDIATE** | **RESISTANT** |
| ATCC 19606^a^ | *A. baumannii* | urine | Cefotaxime, Ceftazidime, Ceftriaxone, Imipenem, Meropenem, Gentamicin, Kanamycin, Neomycin, Tobramycin, Netilmicin, Amikacin, Nalidixic Acid, Ciprofloxacin, Tetracycline, Rifampicin, Trimethoprim | - | Ampicillin, Streptomycin, Spectinomycin, Chloramphenicol, Sulfamethoxazole |
| DF1000^b^ | *A. baumannii* | blood^1^ | Tazocin, Ceftazidime, Cefipime, Meropenem, Amikacin, Gentamicin, Tobramycin, Ciprofloxacin, Cotrimoxazole | - | - |
| DF1001^b^ | *A. baumannii* | blood^1^ | Ciprofloxacin, Gentamicin | - | - |
| DF1008^b^ | *A. baumannii* | blood | Tazocin, Ceftazidime, Cefipime, Meropenem, Amikacin, Gentamicin, Tobramycin, Ciprofloxacin, Cotrimoxazole, Imipenem, Levofloxacin, Doxycycline | - | - |
| DF1009^b^ | *A. baumannii* | blood | Meropenem, Amikacin, Gentamicin, Tobramycin, Ciprofloxacin, Levofloxacin, Cotrimaxazole | - | - |
| DF1010^b^ | *A. baumannii* | blood^3^ | Imipenem, Meroponem, Gentamicin, Tobramycin, Cotrimoxazole, Ciprofloxacin | - | - |
| DF1011^b^ | *A. baumannii* | blood^4^ | Meropenem, Amikacin, Gentamicin, Tobramycin, Ciprofloxacin, Levofloxacin, Cotrimaxazole | - | - |
| DF1012^b^ | *A. baumannii* | blood^4^ | Imipenem, Meroponem, Gentamicin, Tobramycin, Cotrimoxazole, Ciprofloxacin | - | - |
| DF1013^b^ | *A. baumannii* | blood | Ciprofloxacin, Cotrimoxazole, Gentamicin, Meropenem, Amikacin, Tobramycin | - | - |
| DF1015^b^ | *A. baumannii* | blood^6^ | Imipenem, Meroponem, Gentamicin, Tobramycin, Cotrimoxazole, Ciprofloxacin | - | - |
| DF1016^b^ | *A. baumannii* | blood^7^ | Imipenem, Meroponem, Gentamicin, Tobramycin, Cotrimoxazole, Ciprofloxacin | - | - |
| KR1188^c^ | *A. baumannii* | - | Imipenem, Meroponem, Gentamicin, Tobramycin, Cotrimoxazole, Ciprofloxacin | - | - |
| KR1173^c^ | *A. baumannii* | urine | Colistin, Tobramycin | - | Imipenem, Meroponem, Gentamicin, Cotrimoxazole, Ciprofloxacin, Piperacillin, Tazobactam |
| KR1174^c^ | *A. baumannii* | urine | Imipenem, Meroponem, Gentamicin, Tobramycin, Cotrimoxazole, Ciprofloxacin | - | - |
| KR1175^c^ | *A. baumannii* | urine | Imipenem, Meroponem, Gentamicin, Tobramycin, Cotrimoxazole | Ciprofloxacin | Piperacillin, Tazobactam |
| KR792^c^ | *A. baumannii* | wound, left hand | - | Cotrimoxazole | Imipenem, Meroponem, Gentamicin, Tobramycin, Ciprofloxacin |
| DF1003^b^ | *A. calcoaceticus* | blood^2^ | Co-Amoxiclav, Ciprofloxacin, Cefuroxime, Cotrimoxazole, Gentamicin, Tazocin, Cefoxitin, Aztreonam, Ertapenem, Ampicillin, Cefuroxime Axetil, Cefotaxime, Ceftazidime | - | - |
| DF1004^b^ | *A. calcoaceticus* | blood^2^ | Meropenem, Amikacin, Gentamicin, Tobramycin, Ciprofloxacin, Cotrimaxazole | - | - |
| DF1005^b^ | *A. calcoaceticus* | blood^2^ | Ciprofloxacin, Cotrimoxazole, Gentamicin, Meropenem, Amikacin, Tobramycin | - | - |
| DF1006^b^ | *A. calcoaceticus* | blood^2^ | Ciprofloxacin, Cotrimoxazole, Gentamicin, Tobramycin | - | - |
| KR1189^c^ | *A. calcoaceticus* | - | Imipenem, Meroponem, Gentamicin, Tobramycin, Cotrimoxazole, Ciprofloxacin | - | - |
| DF2000^b^ | *A. ursingii* | blood^5^ | Ciprofloxacin, Cotrimaxazole, Gentamicin, Meropenem, Amikacin, Tobramycin | - | - |
| KR1177^c^ | *A. ursingii* | skin | Imipenem, Meroponem, Gentamicin, Tobramycin, Cotrimoxazole, Ciprofloxacin | - | - |
| KR1178^c^ | *A. ursingii* | external ear (otitis) | Imipenem, Meroponem, Gentamicin, Tobramycin, Cotrimoxazole | Ciprofloxacin | - |
| KR1179^c^ | *A. ursingii* | urine (nephrostomy)^8^ | Imipenem, Meroponem, Gentamicin, Tobramycin, Cotrimoxazole | Ciprofloxacin | Piperacillin, Tazobactam |
| KR1180^c^ | *A. ursingii* | urine (catheter)^8^ | Imipenem, Meroponem, Gentamicin, Tobramycin, Cotrimoxazole | Ciprofloxacin | Piperacillin, Tazobactam |
| KR1181^c^ | *A. ursingii* | urine | Imipenem, Meroponem, Gentamicin, Tobramycin, Cotrimoxazole | Ciprofloxacin | Piperacillin, Tazobactam |
| DF3000^b^ | *A. lwoffii* | blood^3^ | Imipenem, Meroponem, Gentamicin, Tobramycin, Cotrimoxazole, Ciprofloxacin | - | - |
| DF3001^b^ | *A. lwoffii* | - | Imipenem, Meroponem, Gentamicin, Tobramycin, Cotrimoxazole, Ciprofloxacin | - | - |
| DF3002^b^ | *A. lwoffii* | blood^6^ | Imipenem, Meroponem, Gentamicin, Tobramycin, Cotrimoxazole, Ciprofloxacin | - | - |
| KR1182^c^ | *A. lwoffii* | urine | Imipenem, Meroponem, Gentamicin, Tobramycin, Cotrimoxazole, Ciprofloxacin | - | - |
| DF1002^b^ | *A. pittii* | blood | Tazocin, Ceftazidime, Cefipime, Meropenem, Amikacin, Gentamicin, Tobramycin, Ciprofloxacin, Cotrimoxazole, Imipenem, Levofloxacin, Doxycycline | Ticarcillin | - |
| DF1007^b^ | *A. pittii* | blood | Meropenem, Amikacin, Gentamicin, Tobramycin, Ciprofloxacin, Cotrimaxazole | - | - |
| KR1165^c^ | *A. pittii* | sputum (lower resp. tract) | Imipenem, Meroponem, Gentamicin, Tobramycin, Cotrimoxazole, Ciprofloxacin | - | - |
| KR1187^c^ | *A. pittii* | urine | Imipenem, Meroponem, Gentamicin, Tobramycin, Cotrimoxazole, Ciprofloxacin | - | - |
| KR1167^c^ | *A. pittii* | urine | Imipenem, Meroponem, Gentamicin, Tobramycin, Cotrimoxazole | Ciprofloxacin | Piperacillin, Tazobactam |
| KR1168^c^ | *A. pittii* | urine | Imipenem, Meroponem, Gentamicin, Tobramycin, Cotrimoxazole | Ciprofloxacin | Piperacillin, Tazobactam |
| KR1169^c^ | *A. pittii* | urine (nephrostomy) | Imipenem, Meroponem, Gentamicin, Tobramycin, Cotrimoxazole | Ciprofloxacin | Piperacillin, Tazobactam |
| KR1190^c^ | *A. pittii* | urine | Imipenem, Meroponem, Gentamicin, Tobramycin, Cotrimoxazole, Ciprofloxacin | - | - |
| KR1170^c^ | *A. pittii* | urine | Imipenem, Meroponem, Gentamicin, Tobramycin, Cotrimoxazole | Ciprofloxacin | Piperacillin, Tazobactam |
| KR1171^c^ | *A. pittii* | urine (urine catheter) | Imipenem, Meroponem, Gentamicin, Tobramycin, Cotrimoxazole, Ciprofloxacin | - | Piperacillin, Tazobactam |
| KR1172^c^ | *A. pittii* | urine (nephrostomy) | Imipenem, Meroponem, Gentamicin, Tobramycin, Cotrimoxazole | Ciprofloxacin | - |
| KR1183^c^ | *A. johnsonii* | sputum (lower resp. tract) | Imipenem, Meroponem, Gentamicin, Tobramycin, Cotrimoxazole, Ciprofloxacin | - | - |
| KR1184^c^ | *A. johnsonii* | urine | Imipenem, Meroponem, Gentamicin, Tobramycin, Cotrimoxazole, Ciprofloxacin | - | - |
| KR1185^c^ | *A. johnsonii* | - | Imipenem, Meroponem, Gentamicin, Tobramycin, Cotrimoxazole, Ciprofloxacin | - | - |
| KR1186^c^ | *A. johnsonii* | lung, muscle dystrophia | Imipenem, Meroponem, Gentamicin, Tobramycin, Cotrimoxazole, Ciprofloxacin | - | - |
| DF4001^b^ | *A. beijerinckii* | blood^7^ | Tazocin, Ceftazidime, Cefipime, Meropenem, Amikacin, Gentamicin, Tobramycin, Ciprofloxacin, Cotrimoxazole, Imipenem, Levofloxacin, Doxycycline | - | - |
| DF4002^b^ | *A. gyllenbergii* | blood | Ciprofloxacin, Cotrimoxazole, Gentamicin | - | - |
| DF1014^b^ | *A. courvalinii* | blood^5^ | Imipenem, Meroponem, Gentamicin, Tobramycin, Cotrimoxazole, Ciprofloxacin | - | - |
| KR1176^c^ | *A. nosocomialis* | urine | Imipenem, Meroponem, Gentamicin, Tobramycin, Cotrimoxazole, Ciprofloxacin | - | - |

**Suppl. Table 2. List of *Acinetobacter baumannii* mutant strains used in the study.**

| **NUMBER** | **STRAIN** | **CAPSULE CHARACTERISTICS** |
| --- | --- | --- |
| 307-0294 | *A. baumannii* | wild-type; K1 serotype |
| 307.30 | *A. baumannii* isogenic mutant | *ptk* mutant, capsule negative |
| 307.30/pNLAC1::*ptk* | *A. baumannii* complemented isogenic mutant | pNLAC1 plasmid complemented *ptk* mutant, capsule positive |
